# Comparative Analysis on Dielectric Gold and Aluminium Triangular Junctions: Impact of Ionic Strength and Background Electrolyte by pH Variations

**DOI:** 10.1038/s41598-020-63831-w

**Published:** 2020-04-22

**Authors:** Iswary Letchumanan, M. K. Md Arshad, Subash C. B. Gopinath, R. D. A. A. Rajapaksha, S. R. Balakrishnan

**Affiliations:** 1 Institute of Nano Electronic Engineering, Universiti Malaysia Perlis, 01000 Kangar, Perlis Malaysia; 2 School of Microelectronic Engineering, Universiti Malaysia Perlis, Pauh Putra, Arau, 02600 Perlis Malaysia; 3 School of Bioprocess Engineering, Universiti Malaysia Perlis, 02600 Arau, Perlis Malaysia; 4 EFAML, College of Chemistry, Central China Normal University, 152 Luoyu Road, Wuhan, 430079 Hubei P. R. China; 5 Fakulti Kejuruteraan dan Alam Bina, Universiti Sains Islam Malaysia, Bandar Baru Nilai, 71800 Nilai, Negeri Sembilan Malaysia

**Keywords:** Materials science, Nanoscience and technology

## Abstract

Field of generating a surface thin film is emerging broadly in sensing applications to obtain the quick and fast results by forming the high-performance sensors. Incorporation of thin film technologies in sensor development for the better sensing could be a promising way to attain the current requirements. This work predominantly delineates the fabrication of the dielectric sensor using two different sensing materials (Gold and Aluminium). Conventional photolithography was carried out using silicon as a base material and the photo mask of the dielectric sensor was designed by AutoCAD software. The physical characterization of the fabricated sensor was done by Scanning Electron Microscope, Atomic Force Microscope, High Power Microscope and 3D-nano profiler. The electrical characterization was performed using Keithley 6487 picoammeter with a linear sweep voltage of 0 to 2 V at 0.01 V step voltage. By pH scouting, I-V measurements on the bare sensor were carried out, whereby the gold electrodes conducts a least current than aluminium dielectrodes. Comparative analysis with pH scouting reveals that gold electrode is suitable under varied ionic strengths and background electrolytes, whereas aluminium electrodes were affected by the extreme acid (pH 1) and alkali (pH 12) solutions.

## Introduction

Currently, diagnosis is the significant front for the early identification of disease and therapeutics. Numerous methods have been practiced for the sensing purpose, but there were still several pitfalls encountered such as low-accuracy, time-consumption and expensive equipment is needed^1^. For example, the conventional technique was used to determine the disease and its prognosis, for which the trained personnel is mandatory to handle the experimental steps. Furthermore, those methods need to be carried out in appropriate laboratories and time-consuming, makes the incompatible with quick decision requirement^2,3^. The quick and accurate diagnostic method is highly in demand to handle a small sample amount. Therefore, an ultimate sensor with outstanding features like easy-to-use, portable, inexpensive and highly sensitive are to measure a low amount sample in real time, which would have a wide clinical application. Development of biosensor with variety of approaches to increase the sensitivity level for the better detection in medical diagnosis was practiced. An earlier study has been conducted^4^ by using a tri-electrode with junction design for human chorionic gonadotropin detection. The limit of detection obtained was 27.78 pg/ml and justified that narrow nanogap is suitable for a point-of-care diagnosis. Another study was conducted recently^5^ using a dielectric sensor for C-reactive protein (CRP) detection. Gold nanorod was conjugated with a capturing probe to enhance the sensitivity level. The achieved limit of detection was 1 pM, which is lower than the original concentration of CRP in human serum. The current challenge with this sensing system is making a narrow nanogap in order to improve the sensitivity further. However, with narrowing the gap size to the lower nanometer, the system creates the short circuit. Another case study was conducted recently using a gold dielectric electrode for Factor IX (FIX) detection. This study was conducted using anti-FIX as bioreceptor, while FIX as the target for this detection and the detection limit was 100 pM. While the specific attention was narrowed down by implementing thin film technology to design novel nanosensors^6^. Thin film materials are unique due to their nano- or micro-dimensions nature followed by a superior electrical conductivity for quick and accurate outcome, which would easier for early detection system in the medical field^7^.

Nanotechnology is a wide field with a great potential to built-up a new era for the sensing applications^8–13^. Generally, nanotechnology is strongly associated with a thin film production technology. In current arena of research, innovatively designed thin film is spiking-out more rapidly due to the highly sensitive with fast response tools, which are drastically needed in the field of biomedicine and analytical sciences^14^. The reason for this demand is due to the outstanding unique features of the thin film^15^. Superlative surface area to volume/ratio, charges on the surface, electrical conductivity and nano-structure and dimension promotes the significant advantages for the hybridization of biological molecules on the active surface area for the early-stage detection as primitive strategy in the biomedical field^15^. Whereby, the current work is presenting the comparative study between gold and aluminium as the transducer material^16–18^.

Gold as a transducer material has crucial characteristics, it can generate sensitive and selective electrochemical systems^19^. Gold has been broadly utilized in nanomedicine for the diagnosing purpose due to the outstanding merits, such as easy production method and fabrication process, chemically stable, and holds a great catalytic activity which can heading towards the formation of better biosensor platforms^20–22^. In the biosensing application, gold is a promising material and plays a tremendously role for the better and high-performance sensing by acting as electroactive intermediates^23^. This is because of the better conductivity and capable of providing a biocompatible surface for the immobilization of biomolecules in a better arrangement^24^. Additionally, this arrangement may increase the affinity of antigen-antibody binding^25,26^. Besides, gold material can assist a rapid electron-transfer which forms one-way electron movement between two electrodes.

On the other hand, aluminium thin film based sensor has possessed a great advancement in nanosensing platforms and demanded in the biomedical field due to the high-biocompatibility, good electric conductor and a versatile nature of the materials^27^. Aluminium is widely known as an inexpensive material for the fabrication of biosensing surface. Further, aluminium can be easily manufactured and it also displays great optical and electrical characteristics. In the biosensor, aluminium utilization seems to be not preferable because of the corrosion due to oxidation and pitting problems^28^. Other than oxidation matter, aluminium is apparently unstable in acidic and alkaline conditions, which can be validated using the outcome of sensor’s performance at different pH. Aluminium is unstable in highly acidic and alkaline condition thus incompatible for the biomolecular analysis or other entities detection with pH at extreme acidic and alkaline. Furthermore, researchers are also considering aluminium as sensing material due to cheaper and easier availability. Therefore to get more insight on corroding and cheap material, comparison study with gold is compulsory to monitor whether the capability is tally or lesser than gold, as gold is an outstanding material in the biosensor markets. Usually to avoid corroding of the aluminium, a metallic layer such as, zinc oxide, titanium oxide has been overlaid. Researchers used these oxide materials and further facilitated the ideal chemical functionalization processes. Moreover, it is easily oxidized and resulting in the oxide layer formation. Aluminium is a less competitive biosensing material compared with gold which has been extensively studied. Concerning the novelty of this manuscript gold can be used because research proves that only micro-areas will be covered with oxygen during the oxidation process and no continuous coating around the gold electrode. Moreover, only 10% of oxygen^29^ will be found in the analyzed micro-areas. Hence, gold electrode can be a promising transducer in biosensing platform. Despite, a rapidly growing biosensor application is highly demanding a facile and cheap material for the development of outstanding biodevices, which can be aided by aluminium or gold electrodes and their differences can be considered (Table 1).

**Table 1:** Characteristic comparison between gold and aluminium as a material for biosensor development.

Properties	Gold	Aluminium
Electrical conductivity	Good in electrical conductivity and able to enhance the sensing performance	
Production cost	Expensive	Low cost and easy to manufacture
Availability	In demand	Easily available
Material degradation	No material degradation	Corrosion and pitting
Optical properties	Good absorption and scattering properties	Good plasmonic properties
Oxidation	Difficult to oxidize	Easily oxidized

**Figure 1:**
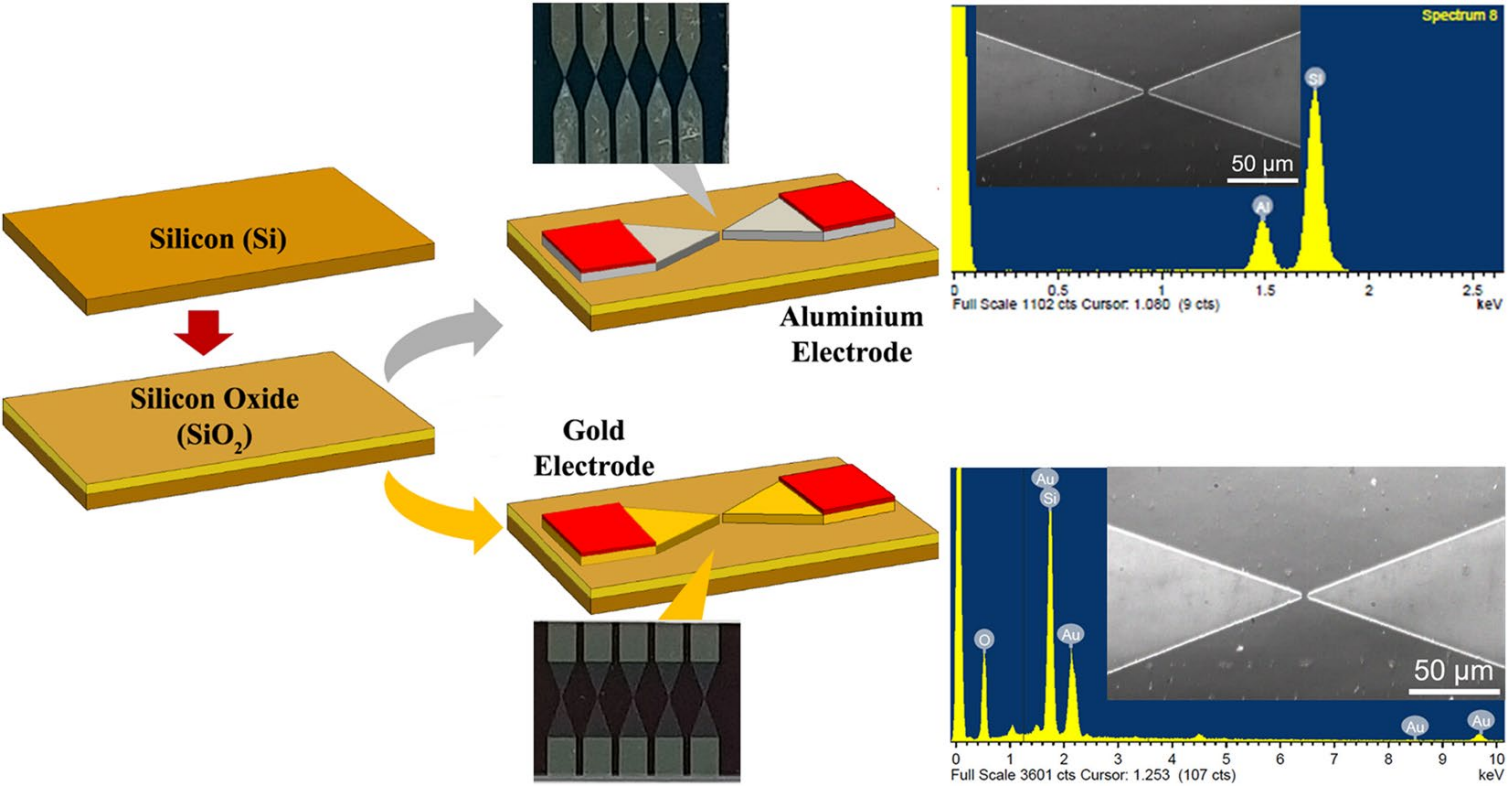
Brief illustration on fabrication flow for both gold and aluminium dielectrics. All the steps are similar except the electrode material. The final design of dielectric electrode was photographed and presented. EDX data was apparently displayed to justify the element inside the dielectric with different materials. Scanning Electron Microscopy Images were included.

In addition, to design an ideal biosensor for the detection of biomolecules in wet condition the analysis of the electrolyte variation need to be conducted. Generally, an ideal biosensor must be unaffected by the pH variations. This is because to ensure that conducted experiment must display the electrical variation due to the biomolecular interaction but not because of the acid and alkaline ions exist with the biological solution. Hence, analysis between a highly acidic (pH 1) to alkaline (pH 14) is strongly recommended. In current study, the dielectric biosensor was fabricated using two different sensing materials. The dielectric-based biosensor is easy to handle during the biomolecular immobilization between two narrow structured triangles. Furthermore, those advantages lead to a key potential dielectric that can utilize as portable biosensors, and able to detect the biological molecules like RNA, protein, oligonucleotide and aptamer. Dielectric was fabricated using silicon (Si) as the substrate by a conventional photolithography method. After the device was successfully fabricated, physical characterizations by High Power Microscopy (HPM), Atomic Force Microscopy (AFM), 3D nano-profiler and Scanning Electron Microscopy (SEM) were carried out. In addition, I-V measurements were done on a bare dielectric sensor and pH scouting using different ionic solutions and the summarized fabrication process is illustrated in Fig. 1.

## Experimental

Reagents utilized in this study were analytical reagent grade. Silicon wafer, Sulphuric acid and Hydrogen peroxide were obtained from Mallinckrodt Baker (USA). Resist developer (RD6) was acquired from Futurrex, Inc to develop the pattern on the substrate. Aluminium etchant, aluminium and gold coil were procured from Sigma Aldrich (USA) for the etching process. Acetone, hydrochloric acid and nitric acid were from Sigma Aldrich (USA) for removing the photoresist at the last-step of fabrication. Positive photoresist (PR1-2000A) was purchased from Futurrex, Inc. (USA) to coat on the silicon substrate. The ready to use pH buffer solutions for the complete ranges were procured from Hanna Instruments (USA). The high power microscope model Olympus BX51 is with the optional resolutions of 5×, 20×, 150×, 250×, purchased from Olympus (Japan). Atomic Force Microscopy (SPA400-SPI4000) is from Seiko Instruments Inc. (Japan). 3D-Nanoprofiler is a Hawk 3D Optical Surface Profiler, obtained from Pemtron Co. Ltd., (South Korea). Scanning Electron Microscopy (JSM-6010LV) is from JEOL (Japan). Thermal evaporator is from Auto 306 Vacuum Coater, HHV Ltd. (India). Muffle furnace (MLFC-1202) is from Tianjin Taisite Instrument Co. Ltd. (China).

### Cleaning of the wafer

Silicon (Si) substrate which is p-type was used as the initiation material. A proper cleaning method is still highly desired to remove the foreign agents, which in contact with the surface of the substrate. This is to avoid the contamination during the fabrication process, which might modify the measurements and the outcome. Thus, piranha solution was prepared using sulphuric acid and hydrogen peroxide in 3:1 ratio^30^. Both solutions were mixed thoroughly and the wafer was immersed in the solution for 15 minutes mainly to reduce metal and organic contaminants. Later, it was cleaned with 70% ethanol and rinsed by the distilled water^31^.

### Thermal oxidation on the silicon substrate

Silicon wafer undergoes wet oxidation process at a high temperature at 1000 °C for 1 hour was to prepare ~3000 Å thick of oxide layer on the wafer substrate to form silicon dioxide^32^, which acts as the better electrical insulator and barrier material with the impurity deposition. The silicon wafer was oxidized in a furnace by passing water vapour (air) over the sample. In the situation of wet oxidation, water molecules are able to dissociate at a high temperature to form hydroxide ions which penetrate the faster into the silicon layer and forms the silicon dioxide. After the oxidation method the sample was placed inside the dry cabinet to avoid the natural oxidation process. The formation of the oxide layer was measured using F20-UV thin-film analyzer.

### Pattern designing on chrome mask

Initially, the pattern of the dielectric sensor was developed using AutoCAD software. The pattern was printed on the blank photomask. Later, the photomask was pasted on the surface of the chrome glass to transfer the pattern. This chrome glass was fixed under the UV exposure system for the pattern transferring. A silicon dioxide substrate deposited with aluminium thin film was placed in the opposite direction against the chrome glass and exposed to UV light for 10 seconds. Pattern was transferred followed by the developing process by using the resist developer.

### Aluminium/Gold deposition

Significantly, the cleaning method must remove all the dirt on the substrate prior to the fabrication process. Then, the aluminium layer acts as a sensing material with the thickness of ~200 nm was deposited on the silicon dioxide layer. This method was carried out using the thermal evaporator (PVD) vacuum coater. The current applied was 50 mA for 5 minutes while the deposition rate was 5.4 nm/s. Gold deposition was done using the same procedure as aluminium deposition process using a gold coil.

### Gold etchant preparation

Hydrochloric acid and nitric acid were utilized to prepare aqua regia mixture. This mixture was prepared using 3:1 ratio. This preparation was carried out in fume hood due to the unpleasant smell of the mixture after the preparation and to avoid direct contact with the acid solution. Herein, 10 ml of nitric acid was poured into 30 ml of hydrochloric acid and stir accordingly.

### In-depth photolithography

Aluminium deposited silicon dioxide wafer was rinsed with acetone and distilled water prior to the fabrication in order to avoid the contamination issues and also for the gold deposited silicon dioxide wafer. Initially, the wafer was coated with a positive photoresist by spin coating technique^33^. The spin coater was utilized to perform three steps whereby all the steps were set with a balanced rpm and the duration of spinning. In photolithography method, the spin speed was formatted into three steps, whereby step 1 is with 1200 rpm for 10 seconds; step 2 is with 3500 rpm for 20 seconds; followed step 3 by 500 rpm for 10 seconds. Initially, the slow spin is to clean the surface of the sample prior to the photoresist coating. Followed by the photoresist coating at steps 2 and 3, there was a slowed-down with the spinning speed. Then, soft bake proceeded at 90 °C for one minute using a hot plate to remove the moisture content on the silicon dioxide surface^34^. Chrome mask with the dielectric pattern was aligned on the surface of the sample and UV exposure was carried out for 10 seconds^35^. The power of the UV lamp is 200 V which is capable for pattern transmission on the sample. Development step was performed to develop the transferred pattern of dielectric by UV exposure using the resist developer (RD6) which will remove the photoresist at unexposed area followed by a hard bake at 110 °C for 2 minutes to increase the adhesion force between the aluminium and silicon dioxide layer and also between gold and silicon dioxide layers. Hard bake also can remove the unwanted moisture^36^. The fabricated device will be observed under a High Power Microscope (HPM) to ensure the design was well developed, for the smooth flow of current between the electrodes. Aluminium etching was done to remove the unexposed area using aluminium etchant solution for 30 seconds. Whereby, gold etching was carried out using prepared gold etchant. The etching time for gold junction is 10 to 15 seconds due to the strong aqua regia solution. The sample was finally rinsed by acetone to remove the photoresist followed by cleaned with distilled water^37^. To dry the samples, air-blower was used.

## Results and Discussion

### Surface morphological analysis

**Figure 2:**
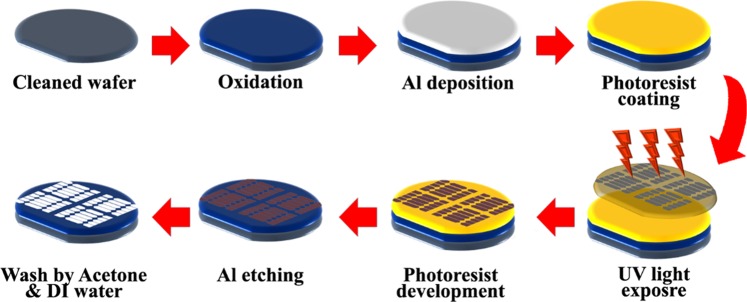
Detailed fabrication step for the development of a dielectric sensor. Initially, starts with wafer cleaning followed by the oxidation process for oxide layer formation. Thin film aluminium/gold deposition as transducer material then photoresist coating. UV exposure was carried out to transfer the exact design of dielectric electrode. Later, etching process was performed followed by washing with acetone then with deionized water. In-depth serial steps involved in the fabrication process were specified.

**Figure 3:**
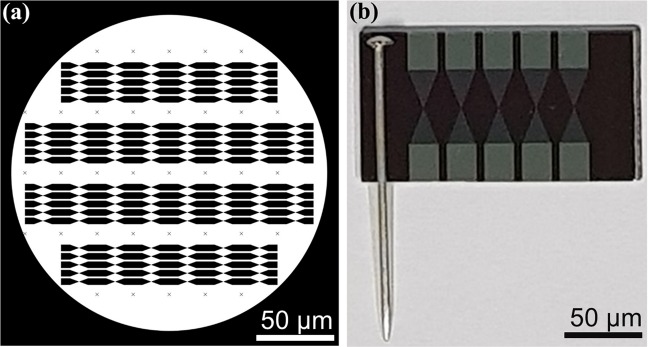
Autocad mediated design (**a**). Proposed dielectric sensor printed on chrome mask; Fabricated dielectric structure for sensing purpose (**b**).

In this study, two types of the dielectric sensor were designed using different materials for sensing purpose. The dielectric was developed such a way that a narrow part of two triangular electrodes facing each at 10 µm gap for the biomolecular immobilization. In this study 10 µm gap size was used at the junction. The current challenge with this sensing system is making a narrow nanogap in order to improve the sensitivity. However, with narrowing the gap size to the lower nanometer, the system creates a short-circuit. To avoid this issue we used 10 µm gap size and our current project is progressing to reduce the gap size. The clear picture of fabrication process is presented in Fig. 2. Energy-dispersive X-ray spectroscopy spectra confirm that the materials as silicon (Si), oxygen (O) and Gold (Au), which were included in the bare gold dielectric and silicon (Si), and aluminium (Al) in the bare aluminium dielectric, without other material contamination as shown in the Fig. 1 inset with SEM images. The end product of the fabrication process was photographed and inserted in Fig. 1 (gold dielectric and aluminium dielectric). Autocad software was utilized to design the proposed dielectric sensor with the proper dimension and it was printed on chrome mask as in Fig. 3a. The successfully fabricated dielectric was diced accordingly to perform the physical and electrical characterization. Figure 3b displays five fabricated dielectric on a substrate.

### Surface physical characterization

**Figure 4:**
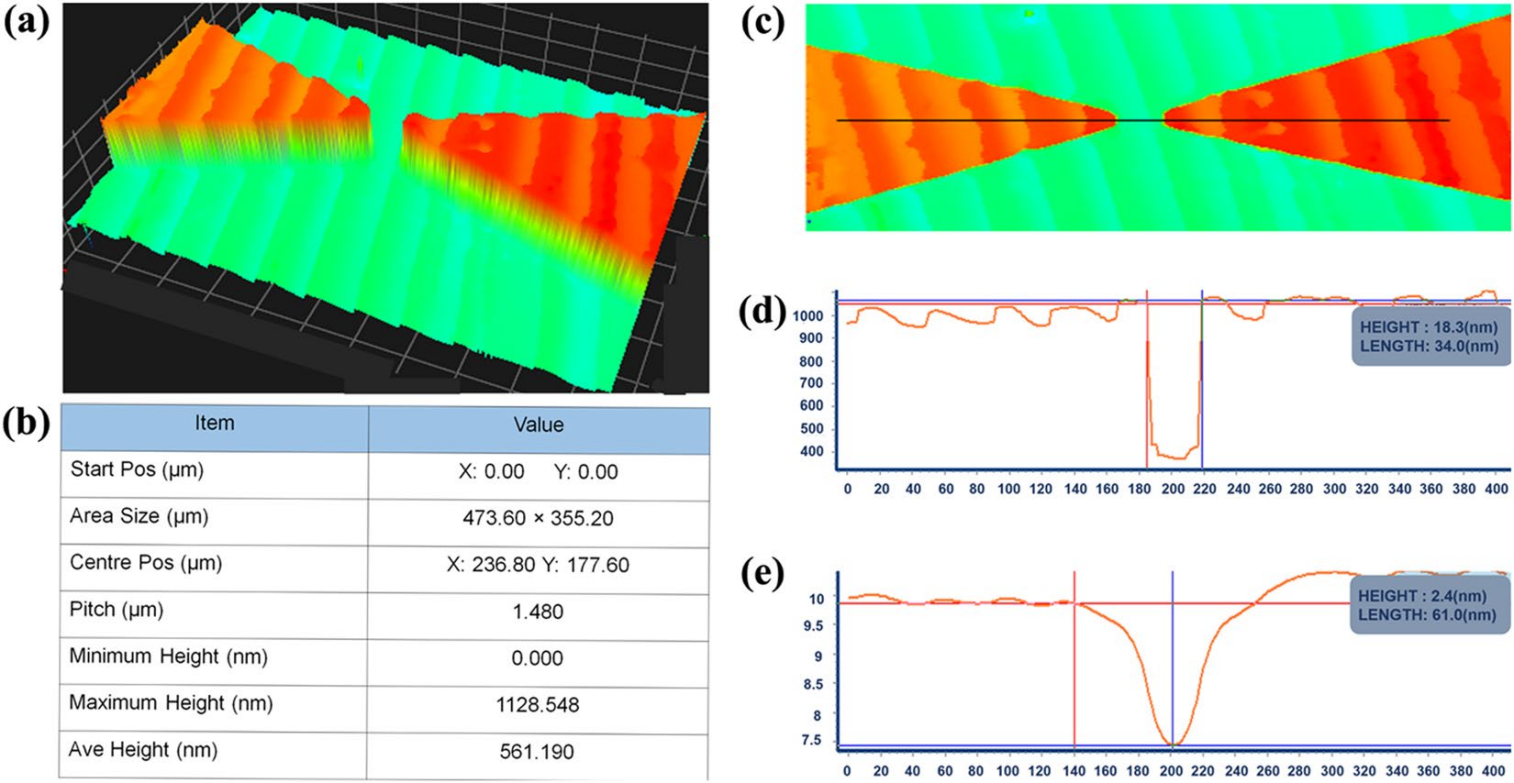
3D nano-profiler imaging. 3D view (**a**): The electrode edges were smooth and sharp as can see in the image; Specification data (**b**): The height, area size and pitch of the fabricated sensor were visibly noted on the table; 2D view (**c**): The gap between the electrodes were fabricated according to the design dimensions.; Surface roughness (**d**) and waviness (**e**) based on the black line shown in 2D image.

Figure 4a illustrates the image of 3D nano-profiler for the sensing surface of the dielectric electrodes. The colour variation represents the height from the bottom level to the top. The maximum height of the surface was 1128.55 nm, while the average height was 561.19 nm as recorded in Fig. 4b. In addition, well-developed electrode edges can be seen clearly in the image. The graph for the surface roughness and waviness of the dielectric electrode was plotted according to the black line as drawn (Fig. 4c–e). The roughness of the dielectric surface is 18.3 nm while the waviness is 2.4 nm.

### Voltammetry measurement on the bare dielectric

**Figure 5:**
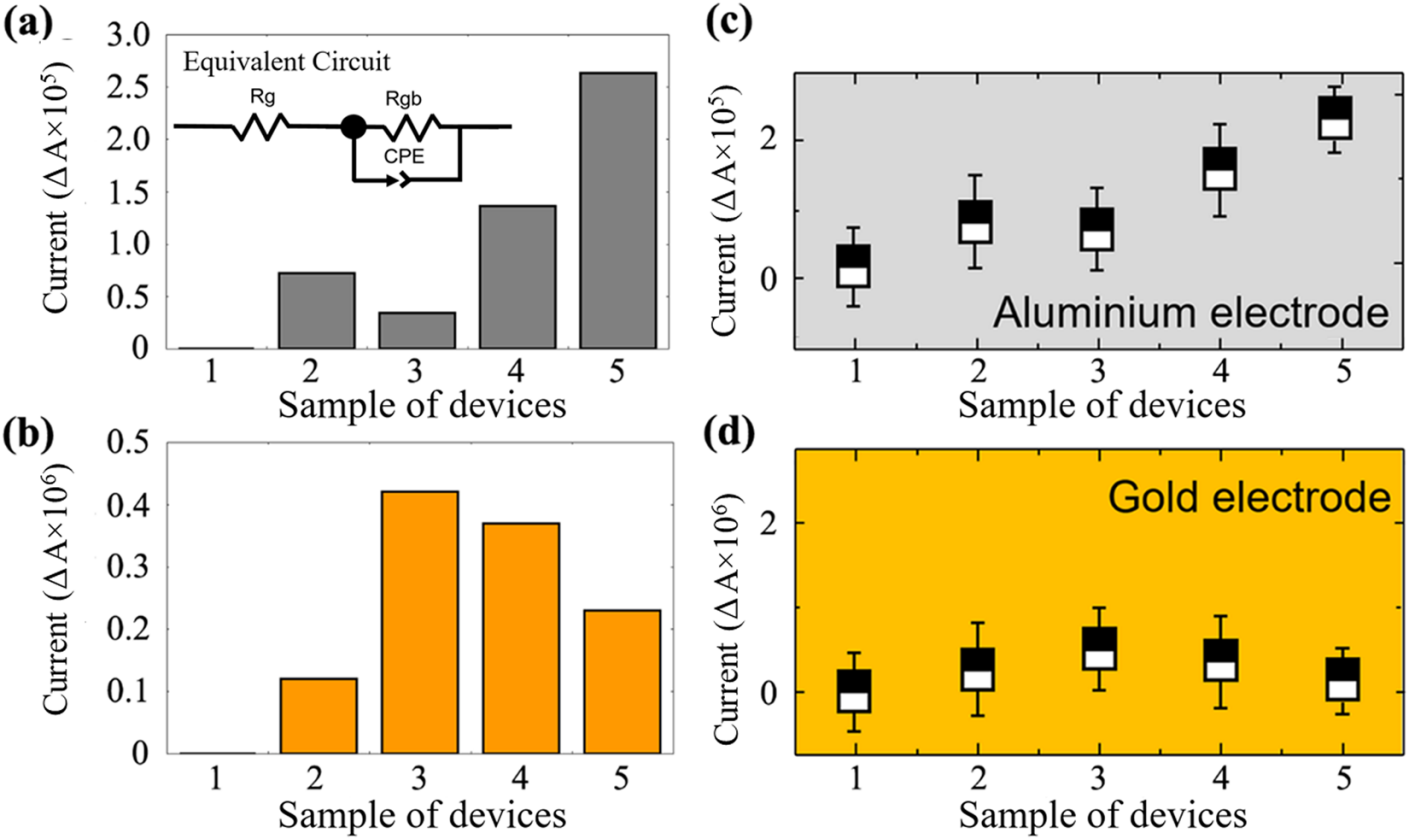
Voltammetry analysis for bare devices. To analyze leakage at the edge of the electrode in order to validate that the sensor was well-fabricated for the better sensing purpose. Aluminium dielectric (**a**), Equivalent circuit (Figure inset); Gold dielectric (**b**); Stability analysis to justify the performance of the dielectric electrode. Aluminium dielectric (**c**); Gold dielectric (**d**).

**Figure 6:**
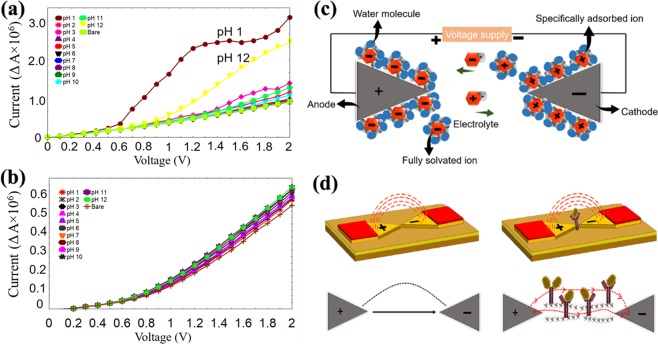
pH scouting. Aluminium dielectric (**a**); Gold dielectric (**b**). pH ranges from 1–12 were tested. Mechanism on the sensing surface. Mobility of the ions between (cathode and anode) electrodes (**c**). Mode of transduction between electrodes (**d**). Dipole moment triggered due to the strong affinity binding between antigen and antibody is clearly shown.

Five different bare devices were electrically characterized to confirm that the dielectric was perfectly developed without any current leakage and to monitor the ability of current conductivity by aluminium-based and gold-based dielectric biosensors. The voltammetry measurements on the bare dielectric were carried out after the sensor was cleaned and dropped with deionized water. This voltammetry measurement was carried out using Keithley 6487 picoammeter with two probes system. Figure 5a shows the current variation among five different bare devices at fixed 2 V. Five different bare devices were electrically characterized to confirm that the dielectric was perfectly developed without any current leakage and to monitor the ability of current conductivity by aluminium-based dielectric.The voltammetry measurements on the bare dielectric were carried out after the sensor was cleaned and dropped with deionized water. This voltammetry measurement was carried out using Keithley 6487 picoammeter with two probe system. The current reading for the devices 1 to 5 reveals about 9.89 × 10^−5^, 8.58 × 10^−5^, 7.55 × 10^−5^, 7.18 × 10^−5^ and 7.90 × 10^−5^ A respectively. Whereby, I-V measurements performed for another five samples which were fabricated using gold as the sensing material to compare the range of current flow between these two different sensing materials. Figure 5b displays the current flow of the five different samples of the bare device at similarly fixed 2 V. The current flow was noted for all the samples. Bare devices from 1-5 reveal the current reading about 2.01 × 10^6^, 2.31 × 10^6^, 1.89 × 10^6^, 2.26 × 10^6^ and 2.12 × 10^6^ A, respectively. Stability analysis was performed to test the performance of the sensor in medical application. Figure 5c,d display that both sensors are holding a similar ability as the presently available high-performance sensors. Figure 5c shows that sensors are holding similar ability as the presently available high-performance sensors with medical applications. Error bars in diagram explain that each sample respectively reveals similar average current reading (Fig. 5a). As seen in Fig. 5a, first sample shows 9.89 × 10^−5^ A and error bar in Fig. 5c shows an average reading of sample 1 at three different measurements with nearly similar value. Hence developed sensor is highly stable throughout and holds a least chance in declining the sensor performance. Thus, obtained results clearly show that aluminium-based dielectric biosensor having a higher current conductivity compared to the gold-based dielectric biosensor. According to the principle of the biosensor, the current flow varies is depending on the attachment of molecules on the surface of the sensor. Though, if the current flow is higher before the functionalization it is difficult to distinguish different types of molecular attachments, while the reading may reveal is due to the combined effect of all ions present in the solution. Normally, when the high concentration of samples was attached on the sensing surface the barrier for the direct current flow increases relatively. This action takes place when there is a high amount of biomolecular interaction on the gap of the sensor, the barrier of direct current flow increases on the electrode surface, which results in increasing the resistance gradually. Figure 6(d) apparently displays the modes of transduction before and after the molecular immobilization. The current flow was smooth without any barrier before the molecular attachment. Whereby, after the immobilization of molecules a high interaction between the analyte and target occur on the sensing surface. Hence, plenty of barriers for the direct current flow were generated and the resistance increases steadily. Formation of molecular complex structure provoked the vibrations, which cause the charge displacement at the dielectric gap. Generated charge displacement with a strong confinement named as a dipole moment^38^. Moreover, the stability analysis shows that both sensors are holding similar ability as the presently available high-performance sensors^39^.

A smaller gap size is able to reduce the electrode polarization effect, and bigger gap is an origin for errors in nanogap electrodes. Therefore, this study would like to suggest reducing the gap size further, because the accumulation of ions (Debye layer) at the narrower gap minimizes the error. Each modification on the surface shows ohmic characteristics and influence the electrode resistivity, based on RT = (ρTlT)/A. Eventually, multiple layers on the surface are resulted in increasing the resistance significantly. It indicates the molecules are trapped in the gap, leading the current flow to be increased and evidences the sensitivity for the changes in chemical reaction. Equivalent Circuit is shown in Fig. 5a as inset. [Rg, Rgp and CPE represent the bulk solution resistance, charge transfer resistance and constant phase element, respectively.

### Voltammetry measurements for pH scouting

In the present study, different pH solutions were utilized to monitor the ions move for the comparison between two different sensing materials. Initially, aluminium based dielectric was undergone pH scouting. In the pH scouting analysis exactly 2 µL of pH buffer has been dropped on the sensor surface using a micropipette, which produces the similar droplet sizes. After each pH solution testing the sensor surface will be rinsed with distilled water to proceed with the following pH solution testing. The Fig. 6a clearly present the I-V readings for all pH solutions (pH 1–12). A huge current variation was monitored for pH 1 and 12, which have extreme acidity and alkalinity levels. Despite, pH solutions except for pH 1 and 12 shows the least difference in comparison among them. The Fig. 6b displays the I-V measurements for the gold-based dielectric sensor. The current variation is very minimum among all the pH solutions (pH 1 to 12), which were tested on the sensor surface. In pH scouting, bare measurement was utilized with deionized water as the control to compare the performance of sensor with and without different pH solutions. In the graph, curve for the bare measurements is lower compared to the pH measurements. This indicates that after the bare sensor was dropped with electrolytes in pH solutions enhance the current flow due to the movement of ions in opposite directions. Positively charged ion will be drawn to anode while negatively charged ion will be drawn to cathode. Herein, electric field will be formed throughout the system.The main objective of pH scouting was to test the impact of ionic strength and background electrolyte at different conditions. It will give the information for the suitability for biomarker detection with various solutions. In order to validate this matter, pH scouting was done to monitor the I-V measurements. Herein, the current variation among all the pH values was calculated using the equation below:

$$\varDelta I={\text{I}}_{\text{n}}-{\text{I}}_{\text{bare}}$$

The equation above can be explained as I_n_ represents the current revealed after the surface was covered with pH solution, while I_bare_ belongs to the current reading of bare device without any attachment. According to the results were acquired for aluminium based dielectric, high acidity and alkalinity (pH 1 and 12) solutions based detection were incompatible. Generally, high level of hydrogen ions (H^+^) can be found in an acidic medium, whereas in an alkaline medium-high level of hydroxide ions (OH^-^) found, which plays a major role in creating the current variation. Commonly, an electrochemical method for the detection comprises of the electrolyte and electrode (metal) with the supplied voltage. Voltage means energy per unit charge needed to transfer the charge from one place to the other for an instance from the cathode to anode. This reaction can be obviously related to the Nernst Equation^40^.

$${\text{E}}_{red}={{\text{E}}_{red}}^{0}-\frac{RT}{zF}\text{In}\,\left[\frac{ared}{aox}\right]$$

The equation can be explained as ‘E’ belongs to the electrode reduction potential, E^0^_*red*_ noted as the standard reduction potential, while ‘R’ is the universal gas constant followed by ‘*T’* is for temperature in Kelvin, ‘z’ fits to the amount of electron was transmitted for each reaction time, ‘F’ for Faraday’s constant while a_red_ and a_ox_ represents the reduced and oxidized molecules in chemical reaction, respectively. Most biomolecules are sensitive to the pH solutions and therefore the detection system must be under the neutral conditions. Generally, pH scouting study has been carried out to monitor the ion movements when encounter the electrolyte solutions. This phenomenon able to produce the electric current is depending on the way of dissociation of ions presence in the solutions. Whereby, with the existence of the electric field the ions can move via the solutions by the supplied voltage. Herein, the negatively charged ions move towards the electrode with depletion of electrons while positively charged ions will move towards the electrode with high amount of electrons. When the neutral atoms or molecules travel through the circuit, isolation and neutralisation will occur on the electrode surface. Hence, different types of pH solution were dropped on the sensor surface to monitor the response of the sensor in purpose for the real detection system. Figure 6(c) clearly shows the movement of electrolytes when anode and cathode were immersed into the solutions contains positively and negatively charged ions. The ions movements through the system are triggered by the voltage supplied. The cations will passage towards the electrode with high amount of electrons while anions will passage towards the electrode lacking the electrons. Thus, the ion movements in opposite direction trigger the formation of the electric current. Herein, curves for pH 1 and 12 are extremely high in current variation compare with the bare sensor. Its shows a huge current conductivity when interact with this particular pH solution. Both pH solutions are sensitive to aluminium based dielectric sensor. Concerning about gold dielectric sensor, obtained graph for gold dielectric electrode displays higher voltages due the evolution of oxygen and this is not the case with aluminium electrode. Because, gold atom was knocked out from the gold electrode by a high energy electron, resulting in fading of gold. Hence, the intensity of electric field is reduced due to the chemical corrosion process. Thus, least variation was observed between the pH curves for gold electrodes. For pH = 1 and 12 cases, the currents are caused by the anodic oxidation of aluminium and reduction by electrolyte solution which leading to the high hydrogen evolution.

Albeit, gold dielectric sensor reveals an outstanding performance towards pH scouting, which indicates this sensor is compatible for all conditions and does not influence by the impact of ionic strength and background eletrolyte. Due to the obtained curve, it shows least current variation among all the pH solutions when compared with bare sensor. The curve validates that pH 1 to 12 solutions are insensitive for gold dielectric sensor and suitable for all applications. According to biosensor principle, easy to distinguish between the attachment of different molecules during the clinical sample detection is due to the least background current variation.

### Detection of FIX using gold-nanogapped sensor

**Figure 7:**
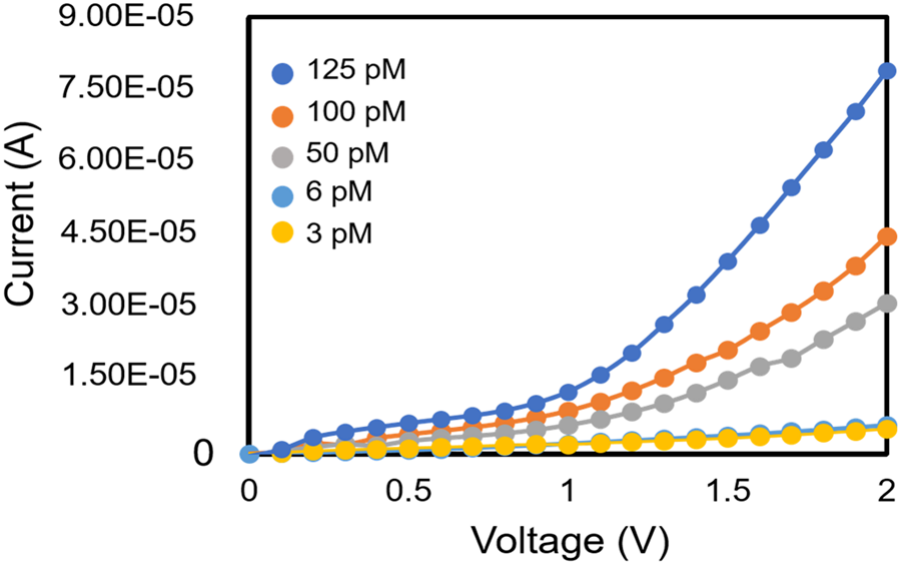
Proof of concept. Graph displays the current flow for dose-dependent interaction of human clotting factor IX on nanogapped sensor.

As the evident, different factor IX (FIX) protein concentrations were titrated on nanogap modified with anti-FIX aptamer. The concentrations from the lower to the higher picomolar range concentrations (3, 6, 50, 100, 125 pM). The current flow was noted to compare the performance between nanogapped sensor and Interdigitated Electrode (IDE) sensor comprises a couple of electrodes, which are arranged in a comb-like fashion^41^. Figure 7 shows the current flow when using the nanogapped sensor. Obviously, the current flow is lower compared to the previous result was obtained using IDE with comb-structured electrodes. According to the principle of the biosensor, the current flow varies is depending on the attachment of molecules on the sensor surface. Though, if the current flow is higher compared to an ideal current (nano- to micro-ampere) it is difficult to distinguish between different types of molecular attachments, while the reading may reveal due to the combined effect of all ions present in the molecules. Normally, when a high concentration of samples is attached on the sensing surface the barrier for the direct current flow increases relatively. This action takes place when there is a high amount of biomolecular interaction on the gap of the sensor, the barrier of direct current flow increases on the electrode surface, which results in increasing the resistance gradually.

## Conclusion

In this study, dielectric sensor with two different sensing materials (gold and aluminium) were independently fabricated using a simple photolithography technique. The p-type silicon substrate was utilized as a base material and the physical characterization was carried out to validate that the sensor was well fabricated with an appropriate dimension. Moreover, the sensing material was uniformly distributed on the surface of the sensor. Electrical characterization was performed on bare devices to confirm that the device is stable when the potential differences were applied. Herein, gold dielectric conduct the least current which is better for detection purpose compared to the aluminium dielectric. Stability analysis for both sensors are found to be good, which can be utilized in various applications are depending on their capability. Nevertheless, pH scouting outcome has revealed that the gold dielectric is insensitive in both acidic and alkali solutions, while aluminium is sensitive in the presence of a maximum concentration of proton or hydroxide ions. A gold dielectric biosensor is a promising tool for the clinical sample detection due to its stability at varied pH.

## Data Availability

The authors declare no supplementary data.
